# Correction: MT-MAG: Accurate and interpretable machine learning for complete or partial taxonomic assignments of metagenomeassembled genomes

**DOI:** 10.1371/journal.pone.0337752

**Published:** 2025-11-25

**Authors:** The PLOS One Staff

## Notice of Republication

This article was republished on November 13, 2025, to correct an error in the title that was introduced during the typesetting process. The publisher apologizes for the errors. Please download this article again to view the correct version. The originally published, uncorrected article and the republished, corrected articles are provided here for reference.

## Supporting information

S1 File Originally published, uncorrected article. (PDF)

S2 File Republished, corrected article. (PDF)
